# PLHPP2 inhibits the stemness of colorectal cancer by inactivating the Nrf2 signaling pathway

**DOI:** 10.7150/jca.65444

**Published:** 2022-02-07

**Authors:** Xiong Yongfu, Zhou He, Huang Xujian, Huang Jiemei, Yang Gang, Zhixiong Lei, Li Jingdong

**Affiliations:** 1 Department of Hepatobiliary Surgery, Affiliated Hospital of North Sichuan Medical College, Nanchong 637007, China.; 2 Department of Thoracic and Hepatobiliary Surgery, Wusheng County People's Hospital, Guangan 638400, China.; 3 Institute of Hepato-Biliary-Pancreatic-Intestinal Disease, North Sichuan Medical College, Nanchong 637007, China.; 4 Research Office of Hepato-Biliary-Pancreatic-Intestinal Disease, Affiliated Hospital of North Sichuan Medical College, Nanchong 637007, China.; 5 Department of Gastrointestinal Surgery, The First Affiliated Hospital of Chongqing Medical University, Chongqing, China.; 6 Department of Gastrointestinal Surgery, Affiliated Hospital of North Sichuan Medical College, Nanchong 637007, China.; 7 Weikang technology, Affiliated Hospital of North Sichuan Medical College, Nanchong 637007, China.

**Keywords:** colorectal cancer, stemness, PHLPP2, Nrf2

## Abstract

Pleckstrin homology (PH) domain leucine-rich repeat protein phosphatase 2 (PHLPP2) is a critical regulator of cellular homeostasis and acts as a tumor suppressor in multiple human cancers. However, its exact biological function in colorectal cancer (CRC) and the underlying molecular mechanism remain poorly understood. The correlation between the transcription and protein abundance of PHLPP2 was analyzed using proteomic and corresponding transcriptional data. Immunohistochemistry was used to validate the protein expression and the role of PHLPP2 in patient prognosis. In addition, a series of experiments *in vitro* and *in vivo* were performed to investigate the underlying molecular mechanism. Immunohistochemical staining of a CRC tissue microarray revealed that PHLPP2 protein expression was significantly downregulated compared to that in adjacent normal tissues. Low expression of PHLPP2 was an independent prognostic risk factor for poor survival. A nomogram established by integrating PHLPP2 expression and traditional clinicopathological factors achieved more reliable prognostic assessment in CRC patients. Additionally, PHLPP2 overexpression suppressed CRC cell migration, invasion and stemness *in vitro* as well as tumorigenesis *in vivo*. Further experiments revealed that upregulation of PHLPP2 increased ROS levels by suppressing the Nrf2-ARE signaling pathway, which inhibited the stemness of CRC cells. Moreover, incubation with sulforaphane, a selective chemical agonist of Nrf2, reversed this inhibitory effect in CRC. PHLPP2 acts as a tumor suppressor gene in CRC by restraining the Nrf2-ARE signaling pathway and increasing ROS levels, affecting the stemness of CRC cells. These anticancer molecular mechanisms indicate PHLLPP2's significant clinical value in prognosis prediction and targeted therapy.

## Introduction

Colorectal cancer (CRC) is one of the most common malignant tumors with a high risk of recurrence and mortality worldwide 1, 2. As a heterogeneous disease, the tumorigenesis of CRC involves a multitude of genetic and epigenetic aberrations 3, 4. However, increasing evidence obtained from multiomics data indicates that the malignant biological behavior of CRC is primarily determined by very few hub genes 5. In the regulatory network, these genes mostly function as a connection to various signaling pathways or control the direction of biological cascade reactions. Thus, screening CRC-related hub genes and further uncovering their molecular mechanism in CRC is critically important for determining tumor markers and therapeutic targets.

In our recently published study, a more effective bioinformatics strategy was implemented to screen hub genes with potential prognostic and therapeutic value in CRC 6. One of the hub genes that we first reported, Pleckstrin homology (PH) domain leucine-rich repeat protein phosphatase 2 (PHLPP2), was found to play a critical role in maintaining the entire aberrant regulatory network. More importantly, mRNA expression of PHLPP2 may represent be an effective biomarker for independently predicting prognosis.

PHLPP2 was identified in a search for phosphatases that dephosphorylate Akt, and emerging evidence in recent years has shown that PHLPP2 not only plays a role in the inhibition of the survival pathway but also in the induction of apoptosis 7. The most well-characterized example of this is in cancer, and recent studies have suggested that PHLPP2 is abnormally expressed in multiple human cancers and may act as a tumor suppressor gene by inhibiting prosurvival signaling pathways, such as PI3K/Akt 8, 9. However, the exact function of PHLPP2 in CRC and its underlying molecular mechanism remain unclear. In addition, as an independent prognostic biomarker that we confirmed in our previous study, whether PHLPP2 can be used to stratify CRC patients through immunohistochemistry (IHC) as effectively as at the mRNA level is unknown. Present methods of gene expression testing usually require frozen tissues, and it is difficult for clinical practice to store enormous surgical resection samples at very low temperatures. Therefore, it is difficult to determine the gene expression of past patients to guide therapy decisions based on aberrant changes at the transcriptional level. However, due to its low cost, convenience and high reproducibility, IHC using formalin-fixed paraffin-embedded (FFPE) specimens is highly available worldwide in hospitals 10. Obviously, IHC may provide a great opportunity for translating our findings into clinical applications.

In this study, we validated that protein expression of PHLPP2 is downregulated, and its IHC features are an independent prognostic risk factor in CRC. In addition, a nomogram established by integrating PHLPP2 levels and traditional clinicopathological factors achieved a more reliable prognostic assessment in individual patients with CRC. Further investigations revealed that PHLPP2 may be involved in the regulation of stemness of CRC cells through the Nrf2-ARE signaling pathway, which leads to changes in malignant biological behavior involving invasion, metastasis and drug resistance. Taken together, our findings, for the first time, demonstrated that PHLPP2 acts as a tumor suppressor gene in CRC by restraining the Nrf2-ARE signaling pathway and increasing ROS levels, thus affecting the stemness of CRC cells. These anticancer molecular mechanisms indicate PHLPP2's significant clinical value in prognosis prediction and targeted therapy.

## Materials and Methods

### Clinical samples

A total of 185 pairs of freshly collected CRC tumor tissues and adjacent normal tissues were collected from patients who underwent surgical resection at The First Affiliated Hospital of Chongqing Medical University. All tissue samples were obtained from patients who were newly diagnosed with CRC and underwent radical surgery without preoperative radiotherapy, chemotherapy or targeted therapy between March 2010 and May 2016. The clinical data are summarized in Supplementary Table S1. Patient inclusion criteria included the following: (1) patients with a pathological diagnosis of CRC; (2) patients with a primary tumor without evidence of distant metastasis before surgery (TNM stage I-III); (3) patients who were treated primarily with surgery; (4) patients with no previous treatment. and (5) patients with complete clinicopathological data and available tissue specimens. Patients were excluded from the study cohorts with the following exclusion criteria: previously received any anti-cancer therapy; impaired heart, lung, liver, or kidney function; previous malignant disease; failure to undergo surgery and the inability to obtain pathological slices. The fresh tissues were fast frozen in liquid nitrogen and kept at -80 °C. All patients were followed up at regular intervals after surgery. Then, a tissue microarray (TMA) was constructed in collaboration with Shanghai Biochip (Shanghai, China) based on our collected CRC samples. The follow-up period was defined as the interval from the date of surgery to the date of death or last follow-up. The latest follow-up was updated in June 2021. All patients were followed up regularly in the outpatient clinic every 3 months during the first year, every 6 months until the fifth year, and then annually. All included subjects had complete follow-up information until death or the latest follow-up date. The acquisition and use of tissue samples passed medical ethics review and informed consent of patients.

### Immunohistochemistry (IHC)

For IHC analysis, the tissue microarray (TMA) specimens were deparaffinized, rehydrated, subjected to antigen retrieval, and endogenous peroxidase was blocked with 3% hydrogen dioxide. Then, the samples were incubated with primary antibodies recognizing human PHLPP2 (anti-PHLPP2, Abcam, USA) at 4 °C for 12 hours, and secondary antibodies (ZSGB-bio, China) were applied for 30 min at room temperature. Staining intensity was classified as 0 (lack of staining), 1 (mild staining), 2 (moderate staining) or 3 (strong staining); the staining percentage was designated as 1 (<25%), 2 (25%-50%), 3 (51%-75%), or 4 (>75%). The final staining score was calculated by multiplying the color intensity and positive cell percentage, and scores ranged from 0 to 12. Scores ≤3 were regarded as low expression, while scores of 4-12 were regarded as high expression.

### Cell culture, plasmid construction and transfection

The human CRC cell lines HT29, HCT116, Caco2, LoVo, SW480 and SW620 were all obtained from the Chinese Academy of Sciences Cell Bank. Cell lines were cultured in DMEM supplemented with 10% fetal bovine serum and penicillin-streptomycin at 37 °C in 5% CO_2_ and 95% air. The PHLPP2 overexpression lentivirus oe-PHLPP2, the PHLPP2 knockdown shRNA lentivirus sh-PHLPP2 and a corresponding negative control (Lv-Cont) were synthesized by GeneChem (Shanghai, China). For lentiviral vector transduction, 293T cells were transfected with the above lentiviral vector for 48 hours. Then, the liquid supernatant was collected. HCT116 and HT29 cells (2×10^5^ cells/well) were seeded into 24‐well plates and cultured in medium for 24 hours. Then, the liquid supernatant was transfected into HCT116 and HT29 cells at a multiplicity of infection of 10. For screening of the transformants, puromycin (4 µg/mL) was added to the medium 96 hours after transfection. The medium was replaced every 2 days for 3 weeks.

### RNA extraction and quantitative real-time reverse transcription PCR (qRT-PCR)

TRIzol reagent (TaKaRa, Japan) was used to extract total RNA from cells. A PrimeScript RT kit (TaKaRa, Japan) was used to synthesize cDNA. qRT-PCR was performed in an ABI7500 Real-time PCR system (Applied Biosystems, Foster City, USA) using the SYBR PreMix ex Taq benchmark (Takala, Dalian, China). The sequences of primers used to amplify PHLPP2, CD44, CD133, EPCAM, GCLC, TXN, GPX2, GCLM, and SRXN1 are summarized in Supplementary Table S2. The relative gene expression was calculated using the ΔCT method with the formula n=2^-(CT targeted gene - CT GAPDH)^.

### Protein extraction, Western blotting analysis, and antibodies

Whole cells were lysed in cell lysate buffer (PMSF:RIPA=1:100, Beyotime, China), and protein concentrations were quantified with a BCA protein quantification kit (Beyotime, China). Proteins were separated by sodium dodecyl sulfate-polyacrylamide gel electrophoresis (SDS-PAGE) and transferred to polyvinylidene fluoride (PVDF) membranes (Millipore, Billerica, MA, USA). The membranes were blocked in fat-free milk and incubated with specific antibodies at 4 °C for 12 hours. The antibodies included anti-PHLPP2 (Abcam, USA), anti-CD133 (Proteintech, USA), anti-CD44 (CST, USA), anti-EPCAM (CST, USA), anti-Nrf2 (CST, USA), and anti-GAPDH (Hangzhou Xianzhi, China). Membranes were then incubated with secondary antibody (ZSGB-bio, China) and detected using a chemiluminescence system (Bio-Rad).

### Flow cytometry

Cells were plated at a density of 2 × 10^5^ cells per well in 12-well plates. After 48 hours, the cells were incubated in the dark at 37 °C using dihydroethidium (DHE) or CD133**^+^** flow antibody. After 30 min, the samples were run on a flow cytometer to detect fluorescence.

### Sphere formation assay

Cells were cultured in 6-well plates, and 50 μl serum-free medium was added to each well at intervals of 48 hours. The spheroids were imaged and quantified under a phase-contract microscope (Leica) after 1 week.

### Drug sensitivity assay

HCT116 cells were inoculated in 96-well plates and cultured in complete media with different concentrations of 5-FU (0.1 μM, 0.5 μM, 5 μM, 10 μM, 100 μM) or oxaliplatin (0.01 μM, 0.05 μM, 0.5 μM, 1 μM, 5 μM). The corresponding absorbance value (A value) of each well in the 96-well plate at 450 nm wavelength was detected. Cell survival rate (%) = (A _administration_-A _blank_)/(A _control_-A_ blank_) × 100%. Taking the concentration as the abscissa and the cell survival rate (%) at different concentrations as the ordinate, a dose-response curve was created, and the half inhibitory concentration (IC_50_) was calculated using Karber's formula.

### Wound healing assay

A total of 10 × 10^5^ cells were inoculated into a 6-well tissue culture plate and cultured to a density of approximately 90%. Scratch wounds were created using 10 μl pipettes, and then the plate was washed twice and cultured with serum-free RPMI 1640 (Gibco, USA). Images were acquired at 0 h and 48 h to determine the migration of cells.

### Transwell migration and invasion Assays

The upper chamber of the Transwell chamber was precoated with Matrigel, 5 × 10^5^ cells suspended in serum-free media were placed into the upper compartment of 8-μm-pore Transwells (BD, USA), and media supplemented with 10% FBS was added into the lower chamber. After culturing at 37 °C for 12-48 hours, the cells were fixed in 4% paraformaldehyde, stained using crystal violet, 5 visual fields (× fields 20) were randomly selected under the microscope for quantification of cells.

### Animal experiments

HCT116 cells stably transfected with oe-PHLPP2 and Lv-Cont were subcutaneously injected into the waist of 4 - 5-week-old female BLAB/nu-c nude mice (5 mice in each group). Tumor volume was measured once a day. Four weeks after injection, the animals were euthanized, and the subcutaneous tumors were measured and weighed. All animal studies were performed with approval from the Institutional Animal Care and Use Committee of the First Affiliated Hospital of Chongqing Medical University.

### Statistic and bioinformatic analysis

Continuous data are presented as the mean±standard deviation (SD). Independent Student's t-test was used for continuous variables. The Mann-Whitney U test was used to compare PHLPP2 levels between groups. Pearson's chi-square test or Fisher's exact test was used to analyze the relationship between PHLPP2 expression and clinical features. Kaplan-Meier analysis with the log-rank test was used to compare patient survival between subgroups. The effect of each variable on survival was determined by Cox multivariate regression analysis. GSEA (https://www.broadinstitute.org/) was used to explore whether the identified gene sets displayed significant differences between the two groups. Statistical significance was determined using the normalized enrichment score (NES) and false discovery rate (FDR). Unless otherwise mentioned, all statistical analyses in this study were performed using R software (version 4.0.3) for Windows, and a p-value ≤ 0.05 was considered to be statistically significant.

## Results

### PHLPP2 mRNA levels positively correlate with protein expression in CRC

**Figure 1:**
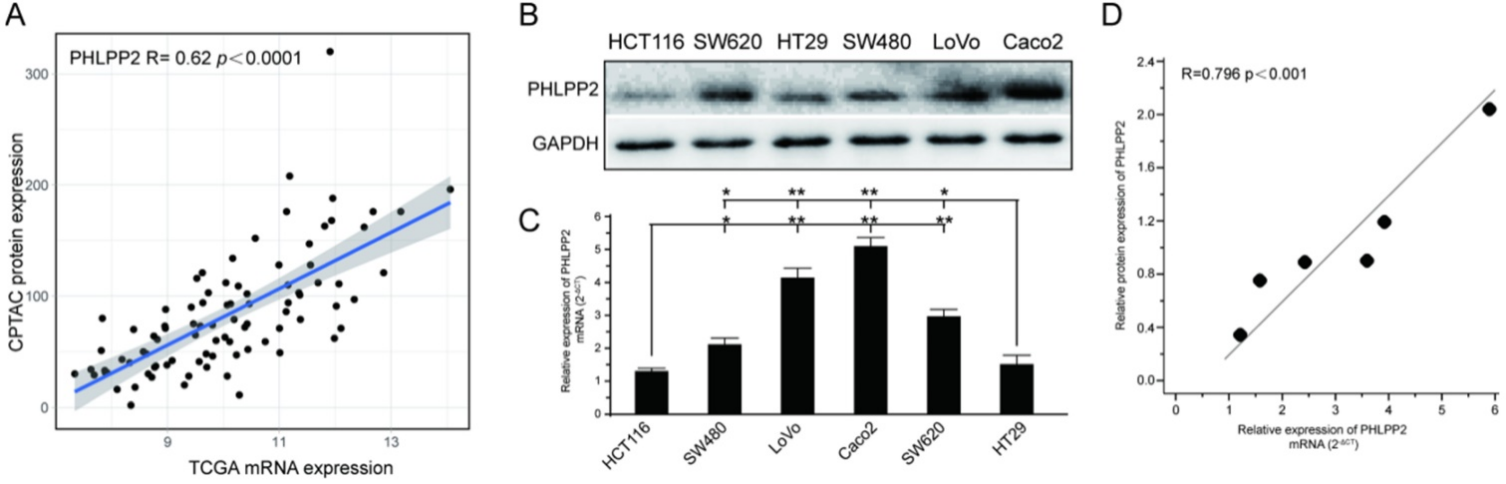
** PHLPP2 mRNA levels positively correlate with protein expression in CRC. (A)** Association analysis between transcript level and protein abundance of PHLPP2 in CRC tissues. **(B)** Protein expression of PHLPP2 in common CRC cell lines. **(C)** Transcriptional expression of PHLPP2 in common CRC cell lines, *P < 0.05; **P < 0.01. **(D)** Correlation between the protein and transcript levels of PHLPP2.

To investigate whether PHLPP2 mRNA expression correlated with protein expression, we downloaded protein expression data from the Clinical Proteomic Tumor Analysis Consortium (CPTAC) data portal (https://proteomics.cancer.gov/data-portal), whose cases overlapped with the TCGA CRC cohort. Finally, after data screening, 90 cases with both mass spectrometry-based protein expression data and transcriptomic data were obtained. Analysis of these paired CPTAC and TCGA datasets revealed that PHLPP2 mRNA and protein expression levels were positively associated in CRC (*R*= 0.62, *P*< 0.0001, Figure 1A). Moreover, the mRNA and protein levels of PHLPP2 in six different CRC cell lines were determined by qRT-PCR and WB. As shown in Figure 1B and 1C, HT29 and HCT116 cells had significantly lower PHLPP2 mRNA and protein expression. Similarly, PHLPP2 mRNA expression was positively correlated with protein abundance (Figure 1D). These results indicate that it is possible to convert our previous findings of PHLPP2 into clinical application through IHC using FFPE tissue sections.

### PHLPP2 is downregulated in CRC and associated with poor prognosis

**Figure 2:**
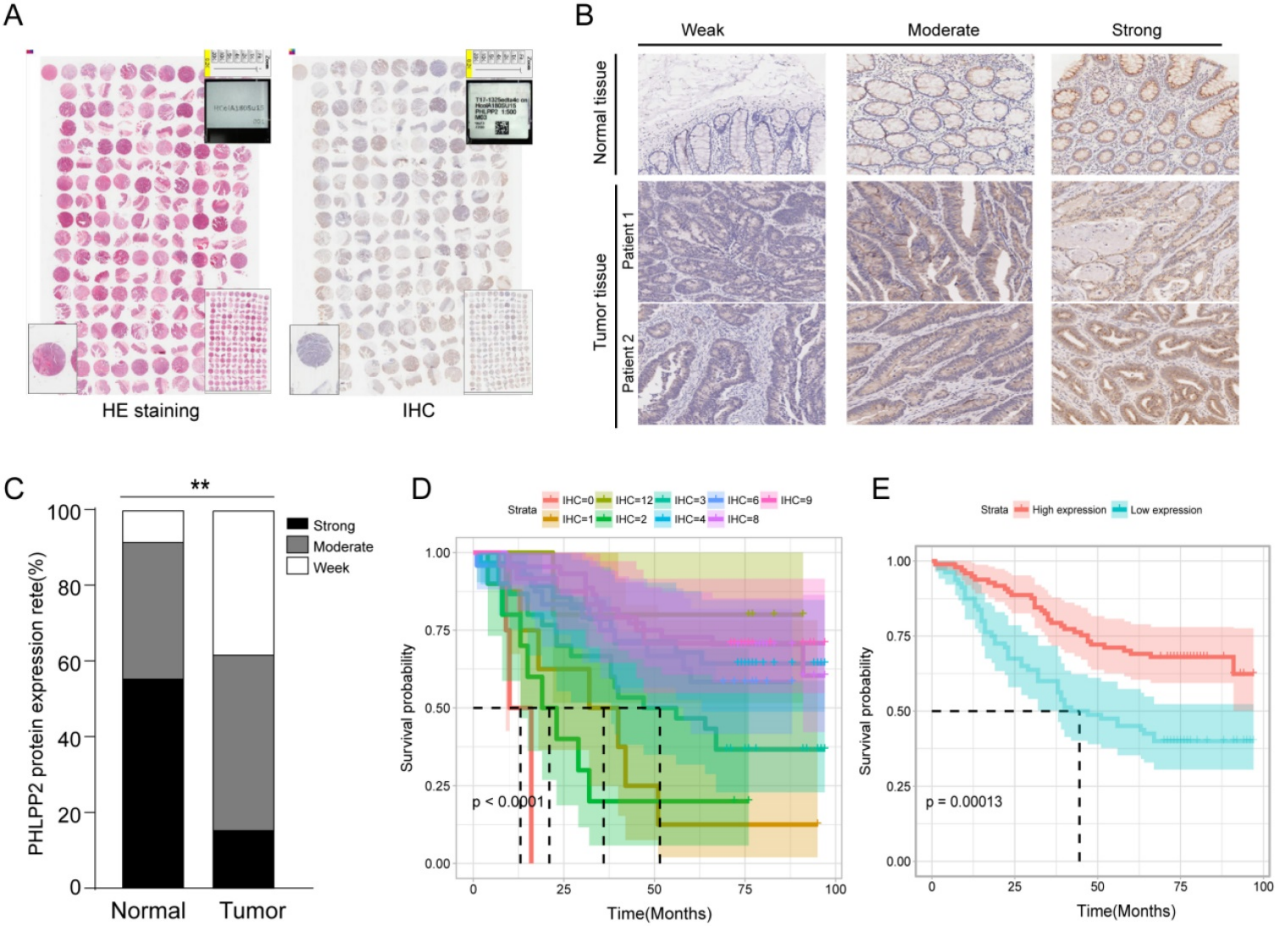
** PHLPP2 is downregulated in CRC and associated with poor prognosis. (A)** The result of immunohistochemical staining of the tissue microarray. **(B)** Representative immunohistochemical expression patterns of PHLPP2 in cancerous and adjacent normal specimens are shown (magnification×200). **(C)** Percentage of cases with different staining intensities of PHLPP2 in the tumor or adjacent normal tissues in the study cohort, **P < 0.01. **(D)** CRC patients were stratified based on the immunoreactive score of PHLPP2 (IRS 0, 1, 2, 3, 4, 6, 8, 9, 12).** (E)** CRC patients were stratified based on expression of PHLPP2 (IRS≤3 low expression, IRS>3 high expression).

To investigate the potential role of PHLPP2 in CRC, we performed IHC on a TMA to analyze expression levels of PHLPP2 protein in 185 pairs of CRC tissues and matched adjacent normal tissues (Figure 2A). As representatively shown in Figure 2B, the immunostaining intensity of PHLPP2 protein was graded on three levels: weak, moderate and strong. The results showed that positive staining of PHLPP2 was observed primarily in the cytoplasm of cancer cells, and high PHLPP2 protein expression was seen in 15.6% (29/185) of CRC tumor samples examined, whereas 54.5% (101/185) of normal tissues exhibited a strong PHLPP2 signal. Overall, these results demonstrate that the expression of PHLPP2 in CRC tissues was significantly downregulated compared to that in adjacent normal tissues (Figure 2C). Correlation with clinical data revealed that expression of PHLPP2 was negatively correlated with age, lymph node metastasis, lymph node positive ratio, tumor differentiation and TNM stage (Supplementary Table S3). In addition, Kaplan-Meier survival analysis indicated that patients with low PHLPP2 expression levels exhibited significantly worse overall survival (Figure 2D-E). Further univariate and multivariate analyses revealed that expression of PHLPP2 was an independent factor in CRC prognostic prediction (Supplementary Table S4). Taken together, these results demonstrate that PPHLPP2 is significantly downregulated in CRC tissues and might be a promising prognostic biomarker in CRC.

### A nomogram integrating PHLPP2 with clinicopathological factors improves individualized prognostic prediction

**Figure 3:**
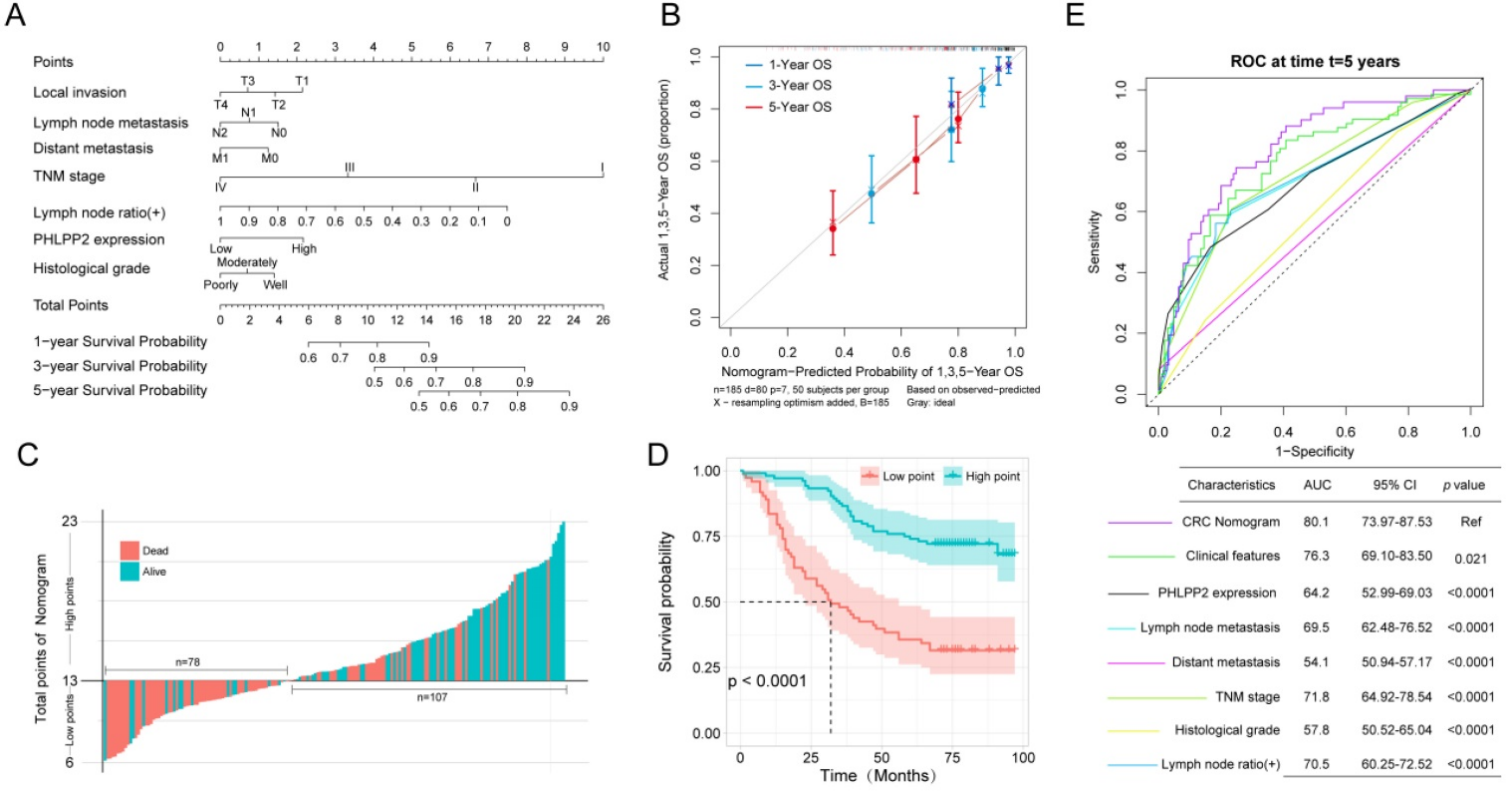
** A nomogram integrating PHLPP2 with clinicopathological factors improves individualized prognosis prediction. (A)** The nomogram, which integrates clinicopathological features and PHLPP2 expression, to assess the overall prognosis of CRC.** (B)** Calibration plot for the nomogram. The dashed line indicates the ideal reference line where predicted probabilities would match the observed proportions. Dashes represent nomogram-predicted probabilities grouped for each of the four quartile groups, along with the respective CIs. **(C-D)** Prognostic evaluation based on nomogram score.** (C)** Nomogram score and survival status of patients. **(D)** Prognostic analysis based on the nomogram score. **(E)** A nomogram was used as a reference to compare the area under the ROC curve with clinicopathological factors and PHLPP2 expression.

Accurately predicting prognosis in patients with CRC is critical to subsequent follow-up and adjacent therapy. To provide clinicians with a quantitative method to generate individualized predictions, we constructed a nomogram that integrated both PHLPP2 and other independent clinicopathological risk factors (Figure 3A). Calibration plots depicted excellent agreement between the nomogram-predicted probabilities and the actual observations of 1-, 3-, and 5-year OS, suggesting appreciable reliability of the nomogram (Figure 3B). Additionally, applying the nomogram stratified CRC patients into distinct risk subgroups with more significant differences in the Kaplan-Meier curves (Figure 3C-D). More importantly, the nomogram achieved the largest area under the ROC curve when compared to traditional clinicopathological factors (Figure 3E). This indicates that the nomogram is superior to any single prognostic factor, and the combination of PHLPP2 and clinicopathological factors further improves the accuracy of individualized prognostic prediction (Figure 3E).

### PHLPP2 may play a major role in regulating the biological characteristics of CRC cells through the Nrf2-ARE pathway

**Figure 4:**
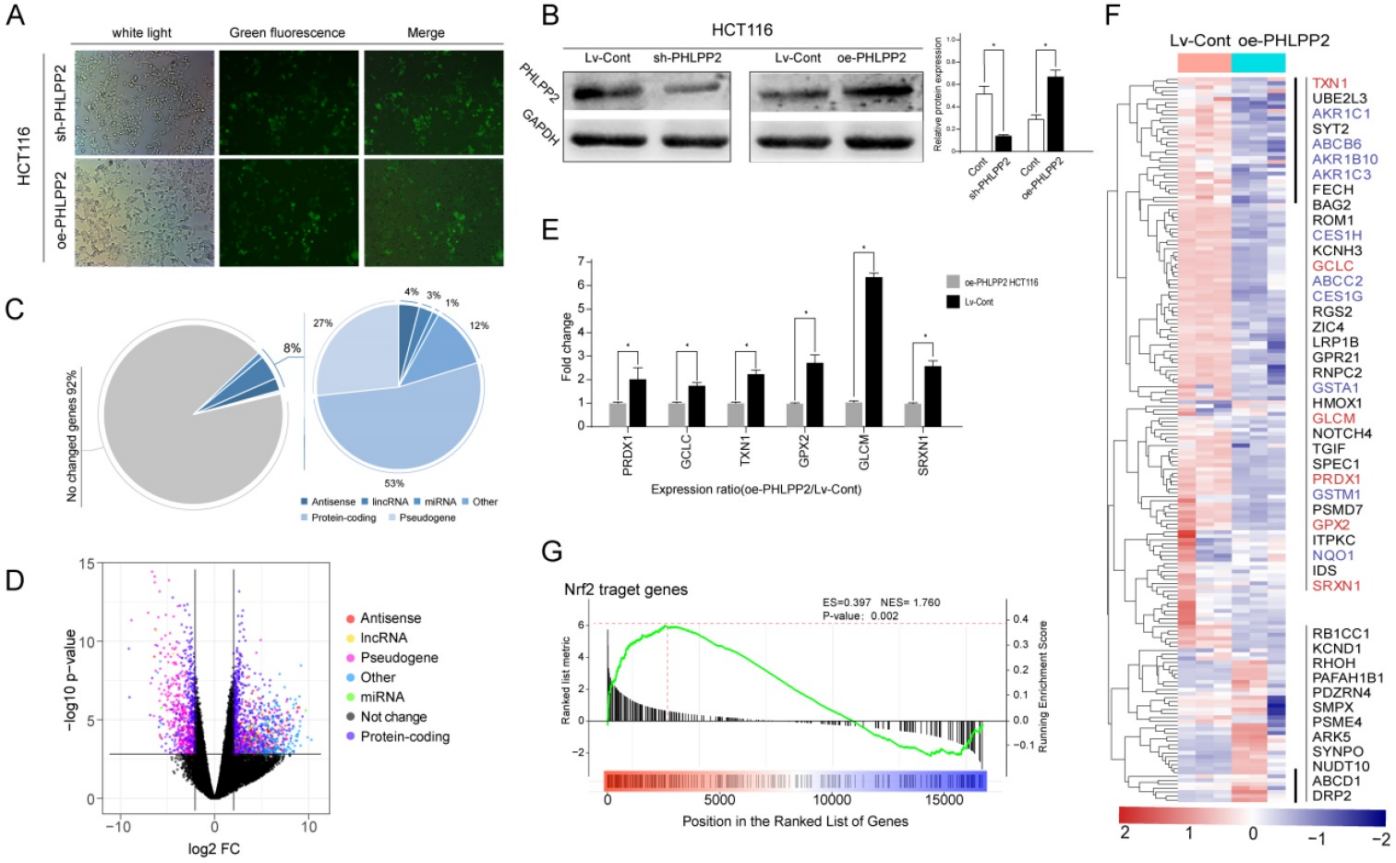
** PHLPP2 may play a major role in regulating the biological characteristics of CRC cells through the Nrf2-ARE pathway. (A)** Transfection efficiency observation with a fluorescence microscope. **(B)** PHLPP2 protein expression was detected by Western blot. **(C)** Genes that may be affected by changes in the expression of PHLPP2. **(D)** Differential expression analysis after interference with PHLPP2.** (E)** PCR validation of key antioxidant genes. **(F)** Heatmap analysis of genes containing antioxidant promoters. **(G)** Enrichment analysis of Nrf2-related genes after PHLPP2 overexpression.

Due to the potential clinical significance of PHLPP2 in CRC, its role cancer biology aroused our attention. To further reveal the molecular mechanism of PHLPP2 in CRC, a series of cell and animal experiments was conducted. First, CRC cell lines HCT-116 and HT-29 with PHLPP2 stable overexpression (oe-PHLPP2) or knockdown (sh-PHLPP2) were established, respectively (Figure 4A). WB confirmed the effectiveness of PHLPP2 overexpression and knockdown (Figure 4B). Subsequently, whole transcriptome sequence (WTS) was performed to explore differentially expressed genes (DEGs) between the oe-PHLPP2 and the Lv-Cont groups. The results suggested that selectively regulating PHLPP2 expression affected approximately 8% of the genes in the entire known transcriptome, of which mRNA accounted for 53%, pseudogenes accounted for 27%, lncRNAs accounted for 3%, and miRNAs accounted for approximately 1% (Figure 4C-D). Intriguingly, during analysis of the DEGs, we found that a large number of antioxidant genes, such as PRDX1, GCLC, GPX2, and GLCM, were significantly overexpressed in the Lv-Cont group, which is visually displayed in the heat map (Figure 4E-F). qRT-PCR analysis confirmed that expression of the abovementioned antioxidant genes was indeed decreased by selectively upregulating PHLPP2 (Figure 4E-F). More importantly, some of the antioxidant-related genes screened by our WTS were found to contain specific promoter regions (5'-GTGACnnnGC-3' sequence). It is well known that genes with antioxidant-related elements (AREs) at their promoter region may combine with the transcription factor Nrf2 (nuclear factor erythroid 2-related factor 2). This process is vitally important for regulating the expression abundance of genes with AREs. Thus, we speculated that the significant clinical value of PHLPP2 was partly based on the molecular mechanism through the Nrf2-ARE pathway. Consistent with speculation, gene set enrichment analysis (GSEA) indicated that the above DEGs were significantly enriched in the Nrf2-ARE pathway (Figure 4G). These results suggest that the effect of PHLPP2 in regulating downstream gene expression may be mediated by the transcription factor Nrf2.

### Overexpression of PHLPP2 increases ROS levels and inhibits the stemness of CRC cells

**Figure 5:**
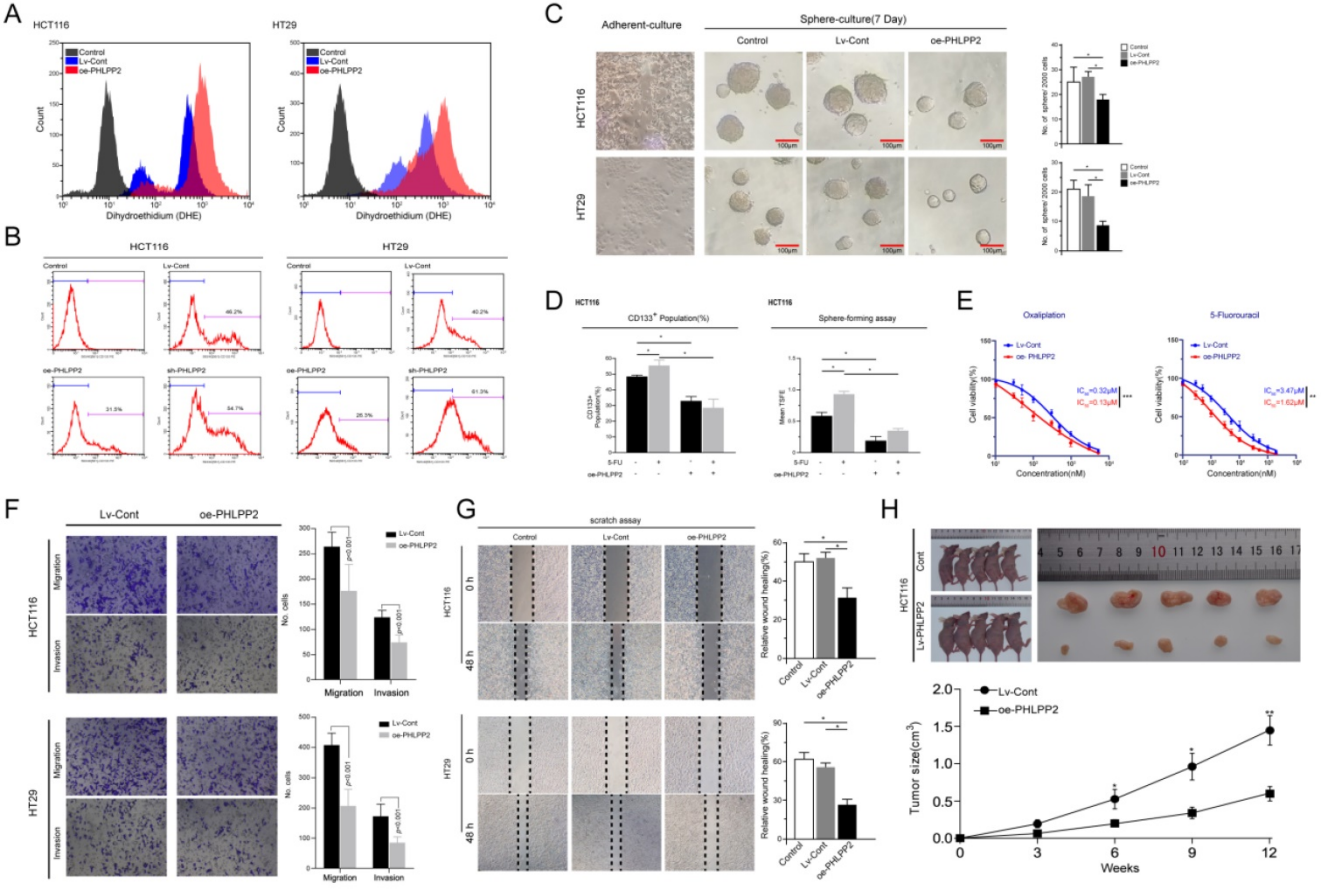
** Overexpression of PHLPP2 increases ROS levels and inhibits the stemness of CRC cells. (A)** The effect of PHLPP2 overexpression on ROS levels in CRC cell lines. **(B)** Flow cytometry was used to detect the proportion of CD133^+^ cells in colon cancer cell lines before and after PHLPP2 knockdown and overexpression. **(C)** Overexpression of PHLPP2 inhibits the formation of stem cell microspheres in CRC cell lines. **(D)** Overexpression of PHLPP2 enhances the effect of 5-FU on CRC stem cells. **(E)** Overexpression of PHLPP2 reduced half lethal concentrations of 5-FU and oxaliplatin. **(F)** The effect of PHLPP2 overexpression on the migration and invasion of CRC cells. **(G)** The effect of PHLPP2 overexpression on the scratch healing ability of colorectal cancer cells. **(H)** Subcutaneous tumorigenesis in nude mice with comparison of tumor dissection and tumor volume. For all, *P < 0.05; **P < 0.01; ***P < 0.001.

Existing evidence recognizes the critical role played by Nrf2 in regulating reactive oxygen species (ROS) 11, 12. In the current study, our bioinformatics analysis further indicated that the gene expression influenced by selectively regulating PHLPP2 was significantly enriched in the Nrf2-ARE pathway. Therefore, we detected ROS levels in the oe-PHLPP2 and Lv-Cont groups by flow cytometry. ROS levels in the oe-PHLPP2 groups were significantly higher than those in the Lv-Cont groups. This trend was consistent between HCT116 and HT29 cells (Figure 5A), indicating that overexpression of PHLPP2 significantly increases ROS levels in CRC cells. These initial results confirm a potential link between PHLPP2, the Nrf2-AER pathway and ROS levels. Our previous studies have revealed that low ROS levels are critical for maintaining the stemness of CRC cells, while increased ROS levels promote the differentiation of CRC stem cells. Thus, we assessed the proportion of CD133^+^ cells in the Lv-Cont, oe-PHLPP2 and sh-PHLPP2 groups in HT29 and HCT116 cell lines. As expected, the proportion of CD133+ cells in the sh-PHLPP2 groups was higher than that in the Lv-Cont groups. However, the proportion of CD133+ cells in the oe-PHLPP2 groups was significantly lower than that in the Lv-Cont group (Figure 5B). These results suggest that maintaining low PHLPP2 expression is important for maintaining stemness. A series of our published studies established that the stemness of CRC cells is associated with various aggressive biological behaviors, including drug resistance, tumorigenicity, invasion and metastasis. Thus, sphere-forming and drug sensitivity assays were used to compare the HT29 and HCT116 cell lines with different PHLPP2 expression levels. The findings indicated that overexpression of PHLPP2 significantly suppressed tumor sphere-forming capability (Figure 5C). In addition, after coculturing with 5-FU, Lv-Cont HCT116 cells were enriched with a higher CD133^+^ subpopulation and possessed a stronger sphere-forming ability, whereas overexpression of PHLPP2 reversed this effect (Figure 5D). We further compared the sensitivity of HCT116 cells to 5-FU and oxaliplatin after overexpression or knockdown of PHLPP2 and observed that overexpression of PHLPP2 significantly reduced the half-lethal concentration (LC50) of 5-FU and oxaliplatin (Figure 5E). We also compared the migratory and invasive capabilities of the Lv-Cont and oe-PHLPP2 groups in HT29 and HCT116 cell lines. Overexpression of PHLPP2 significantly inhibited the migration and invasion of HCT116 and HT29 cells, as detected by Transwell invasion and wound healing assays (Figure 5F-G). Furthermore, the effect of PHLPP2 on tumor growth *in vivo* was analyzed by implanting oe-PHLPP2 or Lv-Cont HCT116 cells into nude mice. As shown in Figure 5h, tumor volumes were significantly decreased in the oe-PHLPP2 group compared to those in the Lv-Cont group. Taken together, these results confirm that PHLPP2 is an important factor in regulating the stemness characteristics of CRC cells.

### PHLPP2 regulates the stemness of CRC cells by targeting Nrf2

**Figure 6:**
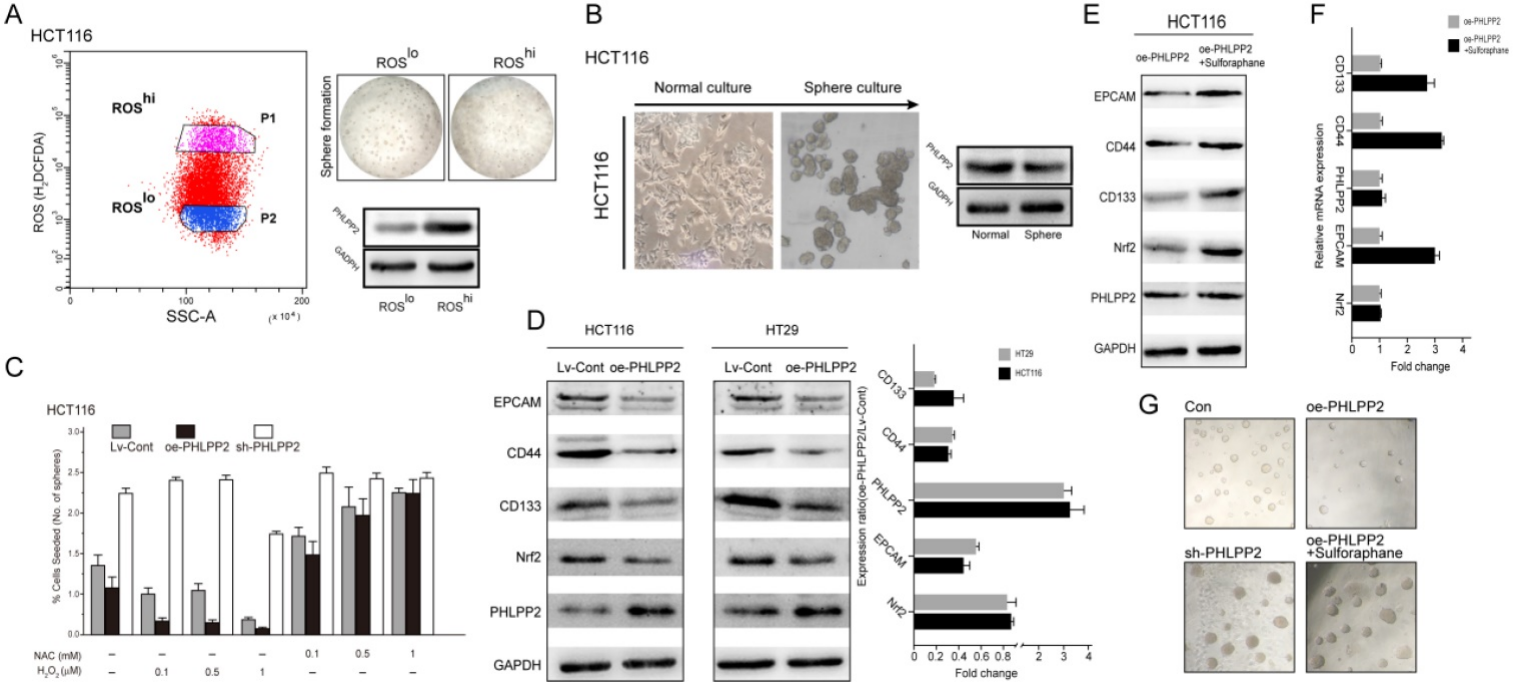
** PHLPP2 regulates the stemness of CRC cells by targeting Nrf2. (A)** HCT116 cells were FACS-sorted based on endogenous levels of ROS using H2DCFDA (left panel), seeded in sphere-forming cultures, and the PHLPP2 protein level was determined (right panel). **(B)** Protein expression of PHLPP2 in HCT116 spheres and normal cultures. **(C)** Effect of ROS modulation on sphere formation of Lv-Cont, oe-PHLPP2 and sh-PHLPP2 cells. Bar graphs represent the number of spheres formed as a percentage response to exogenous NAC and H_2_O_2_ treatment. **(D)** Western blotting was used to detect the expression of stemness marker proteins and Nrf2 in CRC cells before and after overexpression of PHLPP2. The changes in stemness marker genes and Nrf2 in HCT116 and HT29 cells before and after overexpression of PHLPP2 were detected by PCR (oe-PHLPP2/Lv-Cont). **(E)** Western blotting was used to detect the expression of stemness markers before and after overexpression of PHLPP2. **(F)** Expression of stemness marker genes before and after overexpression of PHLPP2 detected by PCR. **(G)** Representative images showing sphere formation of HCT116 cells with Lv-Cont, oe-PHLPP2 and sh-PHLPP2 and Lv-PHLPP2+sulforaphane.

To reveal the underlying mechanism of PHLPP2 in regulating stemness, we first separated high (ROS^hi^)- and low (ROS^lo^)-ROS subpopulations from HCT116 cells and measured their tumor sphere-forming ability. The ROS^lo-^sorted HCT116 cells yielded a higher number and larger tumor spheres with an increased capacity for serial propagation (Figure 6A). Moreover, WB results confirmed that the expression of PHLPP2 protein was significantly lower in ROS^lo^ sorted HCT116 cells (Figure 6A). It is well known that sphere-forming cultures can effectively enrich subpopulations with stem cell properties. Thus, the protein expression of PHLPP2 in tumor spheres obtained from HCT116 cells was determined. Similar to the above results, sphere-forming HCT116 cells had significantly lower PHLPP2 protein levels (Figure 6B). In addition, HCT116 cells were incubated in medium containing various concentrations of hydrogen peroxide (H_2_O_2_) or NAC. As a result, the sh-PHLPP2 group exhibited enhanced resistance to oxidative stress induced by H_2_O_2_ (Figure 6C). These findings demonstrate that PHLPP2 regulates cellular ROS levels, thereby affecting the stemness of CRC cells. Moreover, we detected the expression of representative stemness-related proteins (CD44, CD133, EPCAM) and Nrf2 in HCT116 and HT29 cell lines by WB analysis. The results indicated that CD44, CD133, EPCAM and Nrf2 expression was significantly decreased in the oe-PHLPP2 groups compared to the Lv-Cont groups in both cell lines (Figure 6D). To further confirm whether PHLPP2 regulates the stemness of CRC cells by targeting Nrf2, we conducted a rescue experiment. The results of WB and qRT-PCR confirmed that administration of sulforaphane, a selective chemical agonist of Nrf2, to oe-PHLPP2 HCT-116 cells reversed the decrease in Nrf2 induced by overexpression of PHLPP2 and increased the expression of CD44, CD133, and EPCAM (Figure 6E-F). In addition, administration of sulforaphane also reversed the sphere formation inhibited by high expression of PHLPP2 (Figure 6G). Combining these results, we conclude that PHLPP2 inhibits the stemness of CRC cells by selectively regulating the expression of Nrf2.

## Discussion

A great deal of evidence indicates that colorectal cancer stem cells (CRCSCs) undergo self-renewal and differentiation that are attributed to the metastasis, resistance to therapy, and relapse of CRC, contributing to the poor prognosis of CRC patients^13^, ^14^. Recent studies have begun to shed light on how the maintenance of CRCSC stemness is driven by several crucial pathways, such as Wnt/β-catenin, bone morphogenic protein, TGF-β, and Hh^15^-^17^. In the current study, for the first time, we found that PHLPP2 plays a tumor suppressor role by inhibiting the stemness of CRC cells and demonstrated that Nrf2 is a downstream target of PHLPP2-induced CRC stemness inhibition.

As a pleckstrin homology domain leucine-rich repeat protein phosphatase (PHLPP) isozyme, PHLPP2 is a critical regulator of cellular homeostasis and plays a tumor suppressor role in multiple human cancers 18-20. Previous studies have reported that PHLPP2 may regulate the transcriptional effect of Nrf2 on downstream genes by affecting the transmembrane transport of Nrf2 in hepatocytes 21, 22. In our work, whole transcriptome sequencing results suggested that selectively regulating PHLPP2 expression affects a large number of antioxidant genes, such as PRDX1, GCLC, GPX2, and GLCM, which are pivotal factors in the Nrf2-ARE signaling pathway. Subsequent GSEA indicated that the above DEGs were significantly enriched in the Nrf2-ARE signaling pathway. These results suggest that PHLPP2 may regulate the biological characteristics of CRC cells through the Nrf2-ARE signaling pathway.

Reactive oxygen species (ROS) produced from normal metabolism or external stimuli can cause damage to DNA, proteins and organelles 23. It is widely accepted that low ROS levels are critical for maintaining the stemness of CSCs, while increased ROS levels promote the differentiation of CSCs 24, 25. The Nrf2-ARE signaling pathway is responsible for oxygen radical scavenging, and existing evidence recognizes the critical role played by Nrf2 in regulating ROS 11, 12. Importantly, available research also supports the important role of the Nrf2-ARE signaling pathway in regulating CSC stemness. For example, Jiang et al. demonstrated that Nrf2 inhibition impairs the self-renewal ability of human embryonic stem cells 26. Another study reported that inhibition of the Nrf2-ARE signaling pathway decreased the stemness of bone marrow-derived mesenchymal stem cells 27. Given these findings, we speculated that PHLPP2 may affect CRC cell stemness via the Nrf2-ARE signaling pathway.

CD133 has been recognized as the primary marker for CRCSCs, which also includes CD44 and EPCAM. As expected, overexpression of PHLPP2 significantly increased ROS levels in CRC cells, and the proportion of CD133+ cells in the Lv-PHLPP2 group was significantly lower than that in the Lv-Cont group. Moreover, sphere-forming assays revealed that the levels of ROS affected the self-renewal ability of CRC cells, which is one of the primary characteristics of stemness. More importantly, our data showed that the sh-PHLPP2 group exhibited enhanced resistance to oxidative stress induced by H_2_O_2_. These findings demonstrate that PHLPP2 regulates cellular ROS levels, thereby affecting the stemness of CRC cells. Further study showed that CD133, CD44, EPCAM and Nrf2 expression was significantly decreased in response to overexpression of PHLPP2. Administration of sulforaphane, a selective chemical agonist of Nrf2, concomitantly increased protein levels of CD133, CD44 and EPCAM and reversed the sphere formation inhibited by high expression of PHLPP2. Combining these results, our findings indicate that PHLPP2 inhibits the stemness of CRC cells by selectively regulating the expression of Nrf2.

We discovered that PHLPP2 is downregulated in CRC tissues and has important prognostic value. Mechanistically, our results demonstrated, for the first time, that PHLPP2 regulates the stemness of CRC cells via the Nrf2-ARE signaling pathway. Together, our findings provide evidence for the potential of PHLPP2 as a prognostic biomarker and therapeutic target in CRC.

## Supplementary Material

Supplementary tables.
