# Supplementary material for: PLHPP2 inhibits the stemness of colorectal cancer by inactivating the Nrf2 signaling pathway
Source: J Cancer. 2022 Feb 7;13(4):1313–23. doi: 10.7150/jca.65444 (PMC8899387; doi:10.7150/jca.65444)
Supplement: Supplementary file 1 — Supplementary tables. [file jcav13p1313s1.pdf]

Supplementary Table S1. The clinical features of the 185 CRC patients

Characteristics		No. of patients (%) (n =185)
Age (years)		
	Median(IQR)	66 (27-90)
	Mean(sd)	65.03 (11.17%)
	< 60, n, %	53 (28.6%)
	≥ 60, n, %	131 (70.9%)
	Unknown, n, %	1 (0.05%)
Sex, n, %		
	Female	81 (43.7%)
	Male	104 (56.3%)
Tumor location, n, %		
	Colon	111 (60.0%)
	Rectum	74 (40.0%)
Histological grade, n, %		
	Well differentiated	35 (19.2%)
	Moderately differentiated	116 (62.7%)
	Poorly differentiated	34 (18.1%)
Local invasion, n, %		
	T1–T2	31 (17.0%)
	T3–T4	154 (83.0%)
Lymph node metastasis, n, %		
	N0	115 (62.1%)
	N1	47 (25.8%)
	N2	23 (12.1%)
Distant metastasis, n, %		
	M0	179 (96.7%)
	M1	6 (3.3%)
TNM stage, n, %		
	I	24 (12.9%)
	II	90 (48.4%)
	III	65 (35.1%)
	IV	6 (3.6%)

Lymph node count	
Median(IQR)	6 (1-19)
Mean(sd)	6.69 (3.84)
Lymph node ratio	
Median(IQR)	0 (0-9)
Mean(sd)	1.13 (1.94)
Tumor volume(cm3)	
Median(IQR)	0 (0-9)
Mean(sd)	1.13 (1.94)
Folllow-up(months)	
Median(IQR)	26.25 (1.5-576)
Mean(sd)	43.58 (57.62)
Survival status, n, %	
Alive	104 (56.2%)
Dead	81 (43.8%)

Supplementary Table S2. The primer sequence of genes in this study.

**Gene**	Primer Sequence	**Product Lengths**
PRDX1	R: 5'- CCACGGAGATCATTGCTTTCA -3'<br>F: 5'- AGGTGTATTGACCCATGCTAGAT-3'	150bp
CD44	R: 5'- ATCATCTTGGCATCCCTCTTG -3'<br>F: 5'- CACCATTTCCTGAGACTTGCTG -3'	177bp
CD133	R: 5'- ACAATCCTGTTATGACAAGCCCA -3'<br>F: 5'- GGAAAGTCCTTGTAGACCCAGAAA -3'	126bp
EPCAM	R: 5'- CAAGGACACTGAAATAACCTGCTC -3'<br>F: 5'- CTCCTTCTGAAGTGCAGTCCG -3'	124bp
PHLPP2	R: 5'- CTTACATCTCGTCCTTTGCACT -3'<br>F: 5'- GGTCGTTCAGTAGGTTCCAGTC-3'	135bp
GCLC	R: 5'- GGGAGGAAACCAAGCGCCAT -3'	79bp

	F: 5’- CTTGACGGCGTGGTAGATGT -3’	
TXN	R: 5’- GTGAAGCAGATCGAGAGCAAG -3’<br>F: 5’- CGTGGCTGAGAAGTCAACTACTA -3’	87bp
GPX2	R: 5’- GAATGGGCAGAACGAGCATC -3’<br>F: 5’- CCGGCCCTATGAGGAACTTC -3’	164bp
GCLM	R: 5’- CAAGGACACTGAAATAACCTGCTC -3’<br>F: 5’- CTCCTTCTGAAGTGCAGTCCG -3’	96bp
SRXN1	R: 5’- CAGGGAGGTGACTACTTCTACTC -3’<br>F: 5’- CAGGTACACCCTTAGGTCTGA -3’	126bp

Supplementary Table S3. Association between the expression of PHLPP2 and clinicopathologic characteristics of patients with CRC

Characteristics	No. of patients (%) n =185	PHLPP2 expression Low(%) n=82	PHLPP2 expression High(%) n=103	t/χ2	p value[a]
Age (years)					
Median(IQR)	66 (27-90)	66 (27-90)	61 (30-85)		
Mean±sd	65.03 ±11.17	68.03±10.05	62.61±9.34	3.971	＜0.0001[d]
< 60, n, %	85 (45.9%)	27 (31.7%)	58 (56.3%)		
≥ 60, n, %	99 (53.5%)	55 (68.3%)	44 (42.7%)		0.001[c]
Unknown, n, %	1 (0.06%)	0 (0.00%)	1 (0.10%)		

Sex, n, %					
Female	81 (43.7%)	41 (50.0%)	40 (38.8%)	1.881	0.170[b]
Male	104 (56.3%)	41 (50.0%)	63 (61.2%)		
Tumor location, n, %					
Colon	111 (60.0%)	56 (68.2%)	55 (53.3%)	3.622	0.057[b]
Rectum	74 (40.0%)	26 (31.8%)	48 (46.7%)		
Histological grade, n, %					
Well	35 (19.2%)	15 (17.6%)	20 (19.4%)		
Moderately	116 (62.7%)	44 (53.6%)	72 (69.9%)	9.446	0.008[b]
Poorly	34 (18.1%)	23 (28.8%)	11 (10.7%)		
Local invasion, n, %					

T1–T2	31 (17.0%)	13 (16.2%)	18 (17.4%)	0.009	0.924[b]
T3–T4	154 (83.0%)	69 (83.8%)	85 (82.6%)		
Lymph node metastasis, n, %					
N0	115 (62.1%)	39 (47.5%)	76 (62.1%)		
N1	47 (25.8%)	22 (26.8%)	25 (25.8%)		$<$0.0001[c]
N2	23 (12.1%)	21 (25.7%)	2 (12.1%)		
Distant metastasis, n, %					
M0	179 (96.7%)	78 (95.1%)	101 (98.0%)		0.405[c]
M1	6 (3.3%)	4 (4.9%)	2 (2.0%)		
TNM stage, n, %					
I	24 (12.9%)	8 (9.7%)	16 (15.5%)		0.001[c]

II	90 (48.4%)	30 (36.5%)	60 (58.2%)		
III	65 (35.1%)	40 (48.7%)	25 (24.2%)		
IV	6 (3.6%)	4 (5.1%)	2 (2.1%)		
Lymph node count					
Median(IQR)	6 (1-19)	6 (1-19)	5(1-17)		
Mean(sd)	6.69 (3.84)	7.28±3.84	6.21±3.73	1.915	0.056[d]
Lymph node ratio					
Median(IQR)	0 (0-9)	0.17 (0-9)	0 (0-0.67)		
Mean(sd)	1.13 (1.94)	0.33±0.36	0.06±0.12		
Tumor volume(cm3)					
Median(IQR)	26.25 (1.5-576)	36.00 (4.5-576)	22.57 (1.5-256)		
Mean(sd)	43.58 (57.62)	49.89±67.96	38.54±1.94	7.134	＜0.0001[d]

Folllow-up(months)					
Median(IQR)	66 (1.0-97)	26.25 (1.0-97)	75 (1.0-97)		
Mean(sd)	64.95 (57.62)	49.26±31.20	65.36±24.93	3.901	＜0.0001[d]
Survival status, n, %					
Alive	104 (56.2%)	33 (40.2%)	71 (68.9%)	14.122	＜0.0001[b]
Dead	81 (43.8%)	49 (59.8%)	32 (31.1%)		

[a] Pearson chi-square test([b]), Fisher exact test([c]) and t test([d]) was used for comparison between subgroups. Bold type indicates statistical significance.

Supplementary Table S4. Univariate and multivariate analyses of PHLPP2 expression and overall survival of patients in the study cohort

Variables	Categories	Univariate analysis				Multivariate analysis			
		HR	95% CI		P value	HR	95% CI		P value
Age(≧60/<60years)		1.008	0.987	1.029	0.453				
Sex (Female/male)		0.965	0.621	1.498	0.873				
Tumor site(Colon/Rectal)		0.803	0.51	1.262	0.341				
Local invasion(T1-T2/T3-T4)		1.884	0.942	3.771	0.073				
Lymph node metastasis(ref, N0)						1.618	1.135	2.307	0.008
	N1	2.685	1.623	4.441	0				
	N2	4.659	2.653	8.185	0				
Distant metastasis(M0/M1)		4.435	1.907	10.311	0.001	1.412	1.019	1.956	0.038
TNM stage(ref, I)						1.705	1.19	2.444	0.004
	II	1.749	0.677	4.522	0.248				

III	4.964	1.958	12.583	0.001				
IV	11.493	3.467	38.103	0				
Histological grade(ref, Well)					1.114	0.778	1.597	0.555
Moderately	1.575	0.822	3.018	0.171				
Poorly	2.257	1.065	4.782	0.034				
Lymph node ratio(+)	6.252	3.469	11.269	0	1.193	0.862	1.651	0.288
Tumor volume(cm3,≥Median)	1.426	0.92	2.212	0.113				
PHLPP2(Low/High)	0.426	0.272	0.667	0	0.663	0.476	0.924	0.015
